# Healthcare human resource development in Saudi Arabia: emerging challenges and opportunities—a critical review

**DOI:** 10.1186/s40985-019-0112-4

**Published:** 2019-02-27

**Authors:** Mohammed Khaled Al-Hanawi, Sami A. Khan, Hussein Mohammed Al-Borie

**Affiliations:** 1 Department of Health Services and Hospital Administration, Faculty of Economics and Administration, King Abdulaziz University, Jeddah, Saudi Arabia; 2 Department of Human Resources Management, Faculty of Economics and Administration, King Abdulaziz University, Jeddah, Saudi Arabia

**Keywords:** Healthcare HRD, Saudization, Vision 2030, NTP 2020, Transformation

## Abstract

**Background:**

Saudi Arabia is currently passing through a transformational phase. There is a huge demand on the Saudi healthcare system to provide better healthcare facilities to the rapidly increasing Saudi population, as well as the growing elderly population. Lack of trained healthcare professionals and heavy reliance on foreign workers are significant aspects for policymakers to consider and deal with. It is also important to re-examine the healthcare Human Resource Development (HRD) initiatives so as to provide a huge reserve of healthcare professionals with appropriate learning and competence.

**Method:**

This paper is a critical review based on secondary data collected from various sources including databases, reports, articles, books, government documents and earlier research undertaken in this regard. The paper is an attempt to document and evaluate the various steps suggested and undertaken by the new strategic plan, Vision 2030, and consequently documented in the National Transformation Program (NTP) adopted in April 2016 in the healthcare HRD sphere in Saudi Arabia.

**Results:**

It has been shown that appropriate HRD capacity building needs to be adopted along with the aggressive policy regulation. It is also important to ensure that future health sector investment meets the needs of local healthcare HRD. Saudization and the adoption of the ‘Nitaqat’ program have played an effective role in pushing the Saudization targets in the private sector, and there is a huge scope for the absorption of young trained Saudi boys and girls in the healthcare sector.

**Conclusion:**

Vision 2030 adopted in 2016 is a testimony to a revolutionary step undertaken by the government and that the healthcare sector is also passing through a major shift in its approach and execution. Vision 2030 has come out with a very clear sense of direction to the healthcare sector, and the projected shift from the existing one-third to two-third Saudi-to-foreigner workforce ratio by the year 2030 needs to be adopted carefully to turn the healthcare HRD challenges into opportunities.

## Background

Healthcare facilities have come a long way in the Kingdom of Saudi Arabia (KSA). Starting from the establishment of the first public health department in Mecca by a royal decree in 1925, and Ministry of Health (MOH) in 1950, at present, Saudi Arabia has a total of 487 hospitals providing 72,981 beds, which are around 2.2 beds per 1000 population in the Kingdom [1]. Government has shown its keen commitment in improving the health of the Saudi population and has given high priority in the development of healthcare services at primary, secondary and tertiary levels [2]. In the year 2018 alone, the Saudi government allocated a fund of SAR 146.5 billion (US$1 = SAR 3.75) for health services and social development, which was 15% of the government budgetary expenditures [3]. The KSA healthcare system was ranked 26th (out of 191 countries) by the World Health Organization (WHO), ahead of most of its neighbouring Arabian Gulf countries, such as the United Arab Emirates (27th), Qatar (44th) and Kuwait (45th). It also ranked higher than many other healthcare systems in developed countries such as Canada (30th), Australia (32nd) and the USA (37th) [4].

Despite these achievements, there are still enormous challenges in providing better healthcare services to the fastest growing population of the Saudi Kingdom [5–7]. Between the years 2004 and 2010 (as per census 2010), the annual population growth rate of Saudi Arabia was recorded at 3.2% per annum with a total fertility rate of 3.04. As per the United Nations (UN) projection, Saudi Arabia will have reached 39.8 million by 2025, 54.7 million by 2050 and 61.3 million by 2100 [8]. Increasing life expectancy of people has also added new challenges as to taking care of the elderly population. By 2020, elderly population is expected to grow from approximately 1 million to roughly 2.5 million [9]. This has put a huge burden on the existing infrastructure augmenting the need of a large reserve of trained healthcare professionals. Another major development in this scenario is the adoption of the new strategic plan, Vision 2030, which has added new challenges as well as opportunities for the health sector to attune its strategic imperatives and direction in the coming years.

Vision 2030 adopted in April 2016 identifies its priorities across all economic sectors and serves as a roadmap for the economic development of the KSA. It sought to identify the general directions, policies, goals and objectives of the Kingdom. Accordingly, ministries, institutions and government entities underwent a restructuring process to align themselves to the requirements of the plan. As part of Vision 2030, the National Transformation Program (NTP) 2020 in the year 2016 was adopted, and strategic objectives, key performance indicators (KPIs) and key performance targets (KPTs) were listed for each ministry [10]. In this background, the present paper attempts to both analyse and evaluate the readiness and availability of manpower in healthcare sector to deal with these challenges and priorities.

### Healthcare in Saudi Arabia: an overview

Prior to 1925, healthcare resources in Saudi Arabia were scarce and the infrastructure was relatively weak. In the early 1900s, only three small-sized private hospitals, the Al-Juad Hospital, Al-Kaban Hospital and Al-Shareef Hospital existed in Mecca [11]. The preliminary healthcare service infrastructure had started to develop only after 1925, and it gained momentum after the establishment of the MOH in 1950. The KSA was having hospitals in Mecca, Medina, Taif, Jeddah, Riyadh and Al-Hasa besides a considerable number of clinics which, by the year 1950, increased the number of hospital beds to 1000 with 111 physicians [12]. Human and financial resources were made available in both the public and private sector hospitals for improving the service quality and preventive care. The 1978 Alma Alta Declaration on primary healthcare reforms was also adopted to expand the healthcare delivery across the KSA [13, 14]. Moreover, the healthcare sector was one of the major beneficiaries of public spending during the oil boom of the 2003–2013. The total healthcare spending increased in this period by an average of 9.6% per year over the decade to reach around SAR 84.4 billion in 2013 [15].

Although relatively few models for public healthcare systems can be identified, the ways in which these models are applied vary considerably, with each having its own unique features. However, within this uniqueness, a constant issue exists as to how to raise the standards of healthcare without imposing prohibitive costs on the tax or insurance paying population. One approach is to develop the level of private healthcare effectively so that those who can afford it will opt for it as opposed to public healthcare [16]. Such a scenario may be seen in the KSA as well, where there is a comprehensive public provision of healthcare, but the private system is still, to a certain degree, relatively small and underutilised. The recently adopted Vision 2030 strongly supports the partnership of private and public sectors. However, accepting a deteriorated public system so that those who could afford private healthcare would be more inclined to pay for their treatment is not an acceptable solution either. Therefore, once again, the issue relies heavily on productivity and efficiency. A quite usual and apparently rational approach has been to produce a model of maximum outcomes and measure the gaps in the present situation. However, this will pose the question as to what represents a maximum or optimal outcome. Is it longevity or quality of life? And is it based on preventive or curative medicine? An alternative approach, which has been increasingly adopted internationally, is to measure partial indicators that together lead to the (health) outcome [17]. Examples include lengths of stay, labour hours spent for each unit of care and average costs of treating certain ailments and conditions. Some general aspects that may considerably contribute to improving productivity and efficiency of the healthcare system include the removal of monopolies and vested interests existing within healthcare system [18].

### Key healthcare services indicators in Saudi Arabia

According to the annual statistical book released by the MOH in Saudi Arabia, there has been a significant progress in the key health indicators. Between 1983 and 2017, life expectancy increased from 66 years to 74.9 years. The infant mortality rate (per 1000 live births) declined from 52 to 6.3. The mortality rate for children under 5 years of age also declined from 63 to 8.9. The maternal mortality rate dropped from 3.2 to 1.2. The infection rate for polio was brought to zero in the year 2009. Moreover, there has been a significant improvement in the availability of health resources. At present, Saudi Arabia has 487 hospitals providing 72,981 healthcare beds, which are around 2.2 beds per 1000 population [1]. However, this rate of 2.2 bed is much lower than the world average of 3.3 bed/1000, and with the growing population, even at the existing bed ratio, the Kingdom needs an additional 10,200 beds by 2025 [19].

Nonetheless, it has been noticed that there is a sharp rise in chronic diseases such as obesity, diabetes, hypertension and coronary heart diseases [20]. Saudi Arabia is ranked, by WHO, third in the world for the prevalence of both diabetes and obesity [21, 22]. Obesity is seen to affect both males and females in Saudi Arabia and physical inactivity among urban Saudis as well as smoking, which is a predominant habit in the Kingdom, all help raise the percentage of cancer, diabetes and coronary heart disease [23]. Psychiatric illnesses, osteoporosis and breast cancer are prevalent among Saudi women. Furthermore, depression, anxiety and stress are also predominant [24]. Death caused by road traffic crashes also makes up 4.7% of all mortalities in the KSA, as opposed to the UK, the USA and Australia where such fatalities hardly exceed 1.7%. Similarly, road fatalities in Saudi Arabia have increased over the years. In the last decade, traffic-related deaths, per 100,000 population, increased from 17.4 to 24 where the KSA was found to have the highest share of traffic death rate compared to high-income countries with an accident to death ratio of 32:1, which is found to be the main cause of death among 16–30 year old males in Saudi Arabia [25].

In the present health scenario, with an increasing population growth, which is the highest in the region, there is a huge need for restructuring the health sector, and the McKinsey Global Institute (MGI) report in 2015 also advocated the transformation of the Saudi economy [15]. As a consequence, a new strategic plan for the Kingdom known as Vision 2030 was adopted in the year 2016. The MGI report envisaged to double Saudi Arabia’s GDP and suggested creating as many as six million new jobs for Saudi nationals by 2030. The report identified eight sectors including the health sector, which has potential to generate more than 60% of the growth opportunity.

The MGI report suggested that the Saudi government has to move away from the government-led economic model currently used to a more market-based approach. It envisaged that for better household income, Saudi men and women should jointly participate in workforce. Besides, stakeholders such as households, the private sector and foreign investors need to be involved in the process as well, and the government should adopt a new delivery philosophy while businesses adjust themselves to a more competitive setting and Saudi individuals take more responsibility for themselves [15]. This will lead to the new era of economic growth and employment as well, which will be sustainable in the absence of the oil booms of the past. In that background, the present paper analyses the impact of the new strategic initiatives taken under the new Vision 2030 and the NTP 2020 adopted in the year 2016 on the healthcare sector with an emphasis on the healthcare Human Resource Development (HRD) initiatives.

## Methodology

This paper is a critical review based on the secondary data collected from various sources including databases, specialist organisations’ reports, articles, books, workshop reports, government documents and earlier research done on the Saudi healthcare reforms. This paper attempts to document and evaluate the various steps suggested and undertaken by the new strategic plan initiated in the healthcare HRD sphere in the KSA in recent past. This paper will strive to discuss, analyse and evaluate these initiatives with an emphasis on evaluating the restructuring of the health sector in facilitating the localisation (Saudization) of the workforce employed in this sector.

The documentation and evaluation of the healthcare HRD initiative under the new economic scenario will add to the scarcely existing literature on the healthcare HRD reform in the KSA. This paper can serve as a baseline evaluation of the existing healthcare HRD imperatives in the context of Vision 2030 and can form part of a longitudinal study, which can be carried out after few years. Vision 2030 provides a considerable number of challenges as well as opportunities for the development of human resource policies for the healthcare sector, and an attempt will be made to identify those challenges and opportunities in this regard.

### Healthcare HRD in Saudi Arabia: challenges and opportunities

According to MGI report, the healthcare sector was employing 600,000 employees in Saudi Arabia which constituted about 350,000 healthcare professionals and about 250,000 management and support staff by the end of the year 2014 [15]. In the coming years, the KSA is likely to continue spending heavily on its healthcare facilities as demographic trends show that the country’s population over the age of 65 is expected to double from approximately 3 to 6% over the next decade [9]. This demonstrates that when life expectancy is raised, it can lead to a 25% increase in the requirement of workforce in the health sector during the next decade. Hence, Saudi Arabia needs a significant increase in the supply of the healthcare professionals in order to meet the increasing needs of its population that is both growing and ageing. Currently, every 1000 population are served by 11 healthcare professionals (total population in 2014 = 31 million) in KSA, which is half the average rate (22 healthcare professionals for 1000 population in G20 countries). In order to meet this average benchmark by the year 2030, Saudi Arabia is required to have roughly 710,000 healthcare professionals, which puts an additional demand of 360,000 professionals to what it has employed today [10, 15].

In addition, in order to fulfil the objectives of Vision 2030 and the NTP 2020, a huge number of Saudi nationals will need to be recruited in the sector of healthcare. The localisation of the workforce, referred to as Saudization, is an important aspect of Vision 2030. At present, only 1 out of 3 healthcare professionals is in fact a Saudi citizen. Currently, the number of healthcare graduates who are Saudis may not be adequate enough to replace professionals who retire or simply quit (regardless of filling vacant positions too). Elderly Saudis are expected to have an increasing demand. Thus, the Kingdom is required to have filled no less than a hundred thousand nursing positions by the year 2030. This makes up a net average of 6000 to 7000 new nurses to join the workforce annually. Yet, only 812 nurses who are Saudi citizens graduated in the KSA in the year 2014. Similarly, overspecialization of physicians exists with serious scarcity of family medical practitioners too. In other words, only 5% of the workforce of physicians actually practices the family medicine [15].

Moreover, currently, the private healthcare sector owns 24% of the hospital beds (about 17,622 out of 72,981) and 32% of the hospitals (158 out of 487), and there is a need to expand the private sector participation in the health sector [1]. The NTP 2020 identifies the increase in the private sector participation in a bigger way and the share of spending through alternative financing methods and service provision. It puts its target of 35% expenditure by the private sector to be achieved by the year 2020 [10]. The detailed strategic objectives, KPIs and KPTs, are discussed in the later sections (see Table 1).

**Table 1:** Selective strategic objectives, KPIs and KPTs, adopted under the National Transformational Plan 2020 [10]

Strategic objective	Relevant objectives of vision 2030	Key performance indicators (KPIs)	Baseline	2020 target (KPTs)	Unit	Regional benchmark	International benchmark
1. Increase private sector share of spending through alternative financing methods and service provision	Improve the quality of healthcare services (preventive or therapeutic) Expand privatisation of governmental services Create an attractive environment for both local and international investors and enhance their confidence in our economy	Percentage of private sector contribution in total healthcare spend	25	35	%	37	60
2. Increase the efficient utilisation of available resources	Improve the quality of healthcare services (preventive or therapeutic) Achieve the highest levels of transparency and good governance in all sectors Achieve budgetary balance	Opex for every new inpatient admission	33,000	33,000	SAR	39,000	NA
3. Improve the efficiency and effectiveness of the healthcare sector through the use of information technology and digital transformation	Improve the quality of healthcare services (preventive or therapeutic) Achieve the highest levels of transparency and good governance in all sectors Improve performance, productivity and flexibility of public authorities	Percentage of Saudi citizens who have a unified digital medical record	0	70	%	NA	100
4. Increase training and development both locally and internationally	Improve the quality of healthcare services (preventive or therapeutic) Provide citizens with knowledge and skills to meet the future needs of the labour market	Number of resident Saudi physicians who are enrolled in training programs	2200	4000	Number	NA	NA
5. Increase the attractiveness of nursing and medical support staff as a preferred career path	Improve the quality of healthcare services (preventive or therapeutic) Provide citizens with knowledge and skills to meet the future needs of the labour market	Number of qualified Saudis in the field of nursing and support staff for every 100,000 people	70.2	150	For every 100,000	460	1106
6. Improve healthcare provision before hospitalisation and in the main hospitals (ER and ICU)	Improve the quality of healthcare services (preventive or therapeutic)	Percentage of patients who received emergency or urgent care with medical decision made (admission/transfer/discharge) in less than 4 h in key hospitals	40	75	%	Under Study	95
7. Improve integration and continuity in service provision by developing the primary care	Improve the quality of healthcare services (preventive or therapeutic)	Number of primary healthcare visits per capita	2	4	Number	3.4	7
8. Improve the infrastructure, facility management and safety standards in healthcare facilities	Enhance the liveability of Saudi cities Improve the quality of healthcare services (preventive or therapeutic)	Number of licenced medical facilities (affiliated with the Ministry of Health and private)	40	100	%	100	100
9. Attain acceptable waiting times across all stages of service delivery	Improve the quality of healthcare services (preventive or therapeutic)	Percentage of appointments received in specialised medical disciplines within 4 weeks (average for all specialties in key hospitals)	< 40	70	%	Under study	83
10. Improve governance in the health system in order to enhance accountability with regard to quality issues and patient safety	Improve the quality of healthcare services (preventive or therapeutic)	Percentage of healthcare facilities reporting comprehensive performance and quality measures	10	100	%	NA	100
11. Adopt a national plan for emergency response to public health threats per international standards	Improve the quality of healthcare services (preventive or therapeutic)	WHO emergency preparedness assessment score—average score for Riyadh, Jeddah and Eastern Province	Calculation in progress	4–5	Score	Under study	Under study
12. Identify additional sources of revenues	Expand privatisation of governmental services Achieve budgetary balance	Total revenue generated from private sector for utilising government health resources	0.3	4	SAR billion	NA	NA
13. Improve public health services with focus on obesity and smoking	Improve the quality of healthcare services (preventive or therapeutic)	Increase in percentage of smoking incidence	Calculation in progress	Reduce by 2% from baseline	%	12.5	10.5
		Increase in percentage of obesity incidence	Calculation in progress	Reduce by 1% from baseline	%	19.4	5
14. Improve the quality of life and healthcare service provided to patients outside hospitals	Improve the quality of healthcare services (preventive or therapeutic)	The percentage of patients who get health care after critical care and long-term hospitalisation within 4 weeks	25	50	%	NA	65
15. Improve quality and safety principles as well as skills of service providers	Improve the quality of healthcare services (preventive or therapeutic)	Percentage of hospitals that meet the US median for patient safety culture	10	50	%	NA	50

Generally, with regard to the challenges of the transformational change adopted in the KSA, the MGI report encourages increased labour participation, especially from Saudi women and young people. It advocates developing the skills of Saudi workers by providing proper education and needed vocational training, as well as improving the mobility and flexibility of labour market for both Saudi and non-Saudi workers, hindering growth in regard to the workforce of the public sector, and increasing the cost of recruiting foreign workforce [15].

In this regard, it highlights two significant challenges. The first is the availability of skilled workforce, which can raise the KSA’s economy to a new level of productivity and efficiency. Indeed, the statistics referred to above in terms of numbers of Saudi workers required in the healthcare sector and necessary increases in the infrastructure and support system as the population increases as well as ages, meaning that there must be significant improvements in the productivity and efficiency of the healthcare system. This also refers to a combination of improved outputs relative to inputs and more cost-effective uses of existing resources. If this is not achieved, the choice will be a deteriorating level of healthcare provision or a health budget that is untenable.

In order to improve and raise the standards of household income and also absorb the demographic youth bulge, both women and men in Saudi Arabia will be required to be fully and equally involved in the workforce. Furthermore, the KSA will have to find ways to counteract the alarming discrepancy between Saudi people’s skills and what the labour market actually requires. The benefits and incentives involving work in the KSA are in conflict with having a truly productive labour force. For instance, workers in the public sector earn, on average, around 70% more than those working in the private sector. Moreover, the benefits of unemployment as well as welfare transfers are reasonably higher than wages. Besides, a good number of employers opt to recruit expatriates lacking adequate skills as opposed to Saudi citizens who naturally demand double or triple the rate demanded by non-Saudis [15].

The second challenge for the KSA economy in general and in providing a better healthcare facilities is creating an economic and controlling setting encouraging business through both transparency and unambiguity. This should warrant the need for building upon the progress achieved in the KSA during the last 15 years, with regard to releasing the economy to foster competition and encourage foreign investors. Furthermore, this involves making multifaceted, controlling environments less complicated, less bureaucratic and easier to manage, through expediting procedures, removing red-tapism, and overcoming the obstacles and opening the road towards a broader, more productive and self-managed private-sector environment.

### Healthcare HRD and Vision 2030 and National Transformation Program

Vision 2030 declares to enhance and appropriately employ the capacity of medical centres and hospitals as well as improve the standards of healthcare services (e.g. therapeutic and preventive). This also indicates that the main focus of the public sector should be on prevention-related care and controlling infection besides urging individuals to first start with primary care. Vision 2030 intends to offer healthcare via public organisations so as to raise its standards and also get ready for the advantages of privatisation in the long run. The vision aims to implement a plan to enhance private medical insurance so individuals can better access needed medical services swiftly without having to wait for long until they could meet the medical professionals and doctors. Furthermore, the vision discusses better training of healthcare professionals to improve treatment for chronic diseases such as heart disease, diabetes and cancer, which all threaten people’s health. Seeing beyond oil, it advocates the diversification of the economy to unleash the capabilities of promising economic sectors and privatise some of the government services in the KSA; one of which is health sector [10].

Among other goals, Vision 2030 envisages to increase the competitiveness (current 25th position to the top 10) on the Global Competitiveness Index and enhance the Foreign Direct Investments flow to 5.7% from 3.8% of GDP. It prescribes to have increased the private sector’s contribution from current 40 to 65% of GDP by 2030. The vision also aims to lower the rate of unemployment in the KSA from existing 11.6 to 7% and raise women’s participation in the workforce from 22 to 30% [10]. The NTP 2020 was also adopted in June 2016; with 15 strategic objectives, 16 KPIs and 16 KPTs for the health sector.

These KPIs and KPTs serve as the dashboard to evaluate the efficacy of the implementation of the NTP. The important strategic objectives of NTP which have a direct and indirect impact on the HRD of the healthcare are as follows: (1) to increase private sector share of spending through alternative financing methods and service provision; (2) to increase the efficient utilisation of available resources; (3) to improve the efficiency and effectiveness of the healthcare sector through the use of information technology and digital transformation; (4) to increase training and development on both a national and international level; (5) to increase the attractiveness of nursing and medical support staff as a preferred career path; (6) to improve the infrastructure, facility management and safety standards in healthcare facilities; (7) to attain acceptable waiting times across all stages of service delivery; (8) to improve governance in the health system in order to enhance accountability with regard to quality issues and patient safety; (9) to improve the quality of life and healthcare service provided to patients outside hospitals; (10) to improve quality and safety principles as well as skills of the service providers [10]. The detailed KPIs and KPTs for these strategic objectives are presented in Table 1.

There is also a need to emphasise and link ‘affluence’ diseases to an appropriate HRD capacity building along with the aggressive policy regulation rather than only increasing funding or number of hospital beds, technology, and medicine. An optimal shift from the existing one-third to a projected two-third Saudi-to-expatriate workforce ratio is likely to have occurred by 2030 through sustained policy approaches—converting challenges into opportunities as envisaged in the NTP 2020. Moreover, there is a scope for ensuring that future healthcare sector investment matches the need for local HRD requirements vis-à-vis job opportunities, especially for nurses, doctors and allied professionals and equally disengaging from costly consultant-level specialisation for hospitals to specialist-level primary healthcare. A launching of public-private partnership models in selection of upcoming institutions for resource sharing including human resources is therefore essential [26]. There is a need to significantly increase the hospital utilisation rate from the current 53%, especially in the rural areas, with a focus on the growth in the local HRD initiative in the family medicine area employing local doctors, nurses and allied staff specialised in family medicine.

In this regard, the KSA faces three critical healthcare reform challenges that can be converted into opportunities by undertaking appropriate investment in this sector: (i) tackling the present under par financing and productivity that the private sector can cover, (ii) having a healthcare labour force not designed to handle the increasing prevalence of non-communicable diseases and (iii) an increasing need for skilled professionals both in the clinical medicine area and healthcare management [15]. If the KSA aims to completely fulfil the objectives explained in the points above, a huge number of Saudi nationals are required to be recruited within the sector. Currently, only one out of three healthcare practitioners is in fact a Saudi citizen [15]. A similar picture, but not as significant, can be seen for allied health specialists, e.g. technicians, assistants and therapists.

It was estimated that if the KSA could make the rate of Saudization, i.e. making workforce more local, double for health specialists (e.g. two thirds), this should generate around 400,000 posts by 2030. Furthermore, around 50,000 management and support posts can also be created. Raising the number of Saudi nationals who become health practitioners needs a declining trend to be reversed. It is similar to exerting a great effort so as to enhance the way well-talented Saudi young people view healthcare posts and also cultivate a positive educational environment at faculties, universities and adequately prepared teaching hospitals.

At present, a huge restriction, besides the limited teaching capacity, lies in the lack of on-the-job on-going training provided for medical assistants in public hospitals. Today, overspecialization of medical practitioners exists with serious scarcity of family medical professionals. With the ageing Saudi population and the heavy load of serious chronic diseases, the Kingdom is bound to move its medical workforce towards primary care. Innovative and creative solutions are also required in terms of the actual obstacles facing medical professionals such as flexible work hours and shift-based schedules. Moreover, the stereotypical perception of healthcare institutions, which are usually seen as being ‘hierarchical’ and not taking nurses into consideration with regard to more advanced or specialised tasks, will indeed need improvement as well. Besides training and staffing, the private sector does have a greatly significant role in the country’s healthcare evolvement [15]. The NTP 2020 discusses increasing the attractiveness of the job for the nursing and medical support staff by providing a well-defined career path and identifies to reach the availability of qualified Saudi in the field of nursing and support staff to 150 (per 100,000 population) from the present 70.2 [10].

At present, private healthcare sector makes up to 24% of hospital beds which is around 17,622 out of 72,981, as well as 32% of the hospitals which is around 158 out of 487. If healthcare private sector needs to develop itself, a clear strategy will be needed as to the provisioning areas which could be available for growth. For instance, operators of private sector can play a great role in appropriately developing service provision in certain aspects such as long-term care, day surgery units, rehabilitation and secondary care hospitals characterised by the limited nature of the care delivered. Also, the state could expand privatising specific other areas such as making the manufacturing of pharmaceuticals as well as healthcare education local. The NTP 2020 proposes to have reached 40% share of pharmaceutical manufacturing in Saudi Arabia from the existing 20% by the year 2020. As a short-term goal, the government has assigned certain selected new facilities scheduled for opening in the coming years to operators of private sector and will also assess the effectiveness of the partnership models of public and private sectors together. It will also be beneficial to guarantee cost neutrality and/or savings for the government spending. From a law-enforced point of view, the state will have to tackle the barriers in terms of the participation of private sector, including legal requirements of ownership by Saudi physicians. Besides, the existing investment barriers would also need to be removed [10].

### Saudization of the healthcare workforce and Nitaqat Program

The increase of foreign workers in Saudi Arabia and the shortage of job opportunities for the Saudi citizens have forced the Saudi government to take swift measures to deal with the challenges of the widespread unemployment and employing locals in place of the expatriate workforce. This awareness has triggered the coining of a new technical term ‘Saudization’, which signifies a revolutionary strategy aiming to properly train Saudi nationals to replace foreign or expatriate workers. The word was created by the government of the KSA in the 1970s, but it only became active within the Saudi economic sphere in 1994. The KSA government implemented the Saudization programme intensively through the sixth development plan (1995–1999), and private sector companies employing more than 20 employees were told, by a royal decree in 1995, to reduce the number of non-Saudis by 5% annually [27].

The Saudization program focussing on the private sector, referred to as Nitaqat Program, was firstly launched in Saudi Arabia in June 2011, and consequently, the Saudization level went from 10% in 2011 to 13% in private sector by the end of 2012. In addition, in the past, the private sector had passively reacted to Saudization, and the actual enforcement of localisation was non-active until the late 1990s [28]. In the healthcare sector, as per the MOH annual report, the foreign or expatriate workers constitute about two thirds of the physicians, nurses and pharmacists employed [1]. The details of employment ratio in the health sector are shown in Table 2.

**Table 2:** Healthcare professionals employed in Saudi Arabia (year 2017) [1]

Types of healthcare professionals	Total number of employees	Percentage of Saudi workforce
Physicians	98,074	29.5
Nurses	185,693	36.7
Pharmacists	28,312	22.2
Allied health personnel	111,861	74.7

The MGI report provides the employment levels of all Saudi citizens and an estimated growth in Saudization, i.e. the share of total employment calculated in the private and the public sectors held by the Saudi nationals. Currently, a positive relationship between wages and Saudization exists, where Saudi citizens take over high-income sectors and expatriate workers control low-income sectors. It is expected that the possible impact of the efforts exerted by the government and the changes of policy can swiftly expedite the process of higher level of Saudization [15]. Similarly, there is likelihood that Saudi citizens may start getting placed in the posts currently filled by the expatriate workers at the dominating low income that expatriate workers currently receive. The rate of unemployment is greatly increasing from the current levels, forcing the state to take action by limiting visas for expatriate workers using a fixed ratio, e.g. 50% for women and 10% for men. In the case of female employment in particular, it endorses that the female share of employment is likely to grow at a very high rate. There is a maximum rate of female employment that is projected for each sector depending on the type of the sector as well as the traditional norms prevailing regarding such work in the KSA. Thereupon, based on the Saudization rate, the need for the number of non-Saudi workers is estimated, assuming the existing ratio of non-Saudi workers to the total non-Saudi population at present [15].

The ‘Nitaqat’ Program was launched to replace the previous Saudization program. Nitaqat (which translates to ‘zones’ or ‘bands’) defines Saudization targets for each firm in the private sector with more than five employees. The present Nitaqat Program divides organisations into six categories namely Platinum, Green (high, medium, low), Yellow and Red, depending on their size and the percentage of Saudization level achieved. Platinum and Green categories represent the highest ratios of Saudi nationals in their organisations, whereas Yellow and Red indicate the lowest ratios of Saudi nationals employed. Thus, based on their performance, it classifies them according to a colour scheme: Red or Yellow refers to non-compliant companies, while compliant companies are referred to as Green, and finally outperforming organisations are referred to using Platinum. Nitaqat was quite different than the previous program to a significant degree. Firstly, the program could be effortlessly supervised by the state. The information and data in terms of the number of Saudi national workers and expatriate workers recruited by a company were collected regularly and systematically using the state’s integrated system of social insurance and foreign visa records. Also, the program of Nitaqat was primarily implemented to set attainable goals and objectives. Nitaqat divided companies into more than 50 different types of business. Moreover, it classified them based on 5 different segments with fixed objectives in each according to the level of Saudization which was already reached by the companies in the given segment. For instance, if the number of employees in a given firm is between 6 and 49, the Saudization requirement could be between 5 and 34% of the total labour force [15].

Unlike previous efforts, it has been noted by the policymakers that ‘Nitaqat has real teeth’. Those companies referred to as being red are successfully stopped from growing due to the restrictions put on them in terms of reissuing more visas for the expatriate workers they are recruiting. However, fewer limitations are imposed on the Yellow companies, and the Platinum ones are compensated with swift and real-time access to expedited government services including smooth visa processing and/or flexible grace periods given after expiration.

## Conclusions

There has been a major shift in the policy direction in Saudi Arabia in the recent past, and the government has taken a very rigorous initiative to balance its expenditure and fiscal deficit. The new Vision 2030 adopted in 2016 is a testimony to revolutionary steps that the government has started to take in all sectors. The healthcare sector is also passing through a major shift in its approach and assumptions. In the healthcare HRD initiatives, the government has chalked out many plans, policies and benchmarks to ascertain the effectiveness of all stakeholders. It has come out with strategic objectives, KPIs and KPTs, which provide a very clear sense of direction to the healthcare sector.

The government is aiming to generate SAR 4 billion from the utilisation of its services by the private sector in the coming 4 years (2016–2020). It plans to have increased the participation of private sector in healthcare expenditure by 10% (25 to 35%) by 2020. There are a number of steps taken by the government, which will surely enhance the larger participation of Saudi workforce in the healthcare sector. The NPT 2020 clearly identifies the training and development, career planning and talent management areas for the healthcare professionals. Saudization of workforce, which for long felt the need to counter the rising unemployment level of Saudi youth, will surely find an answer through these plans. Secondly, an optimal shift from the existing one-third to a projected two-third Saudi-to-foreigner workforce ratio by 2030 through sustained policy approaches has to be adopted carefully, thus converting challenges into opportunities. There is a huge scope for the absorption of young trained Saudi boys and girls in the healthcare sector.

The private sector role in the creation of more medical, nursing and dental colleges and other specialised learning institutions is a challenge as well as an opportunity for the first movers in this sector. There is going to be a huge demand of healthcare professionals, and government and private sector partnership needs to expedite this process by providing more learning and development facilities to counter the rising demand of healthcare professionals. Consequently, the role of private sector in human resource training also needs to be enhanced and redefined in the new context especially for long-term care, rehabilitation services, day surgery units and secondary care hospitals where the complexity of care delivery is limited.

There is a need to adopt appropriate HRD capacity building along with the aggressive policy regulation rather than increased funding or number of hospital beds, technology and medicine. It is also important to ensure that the future health sector investment matches the need for the local healthcare HRD needs. Launching the public-private partnership models in selection of upcoming institutions for resource sharing including human resources will be a real test. However, the policy direction given in the new plan envisages for creating a synergy between all stakeholders and facilitates to give the required push to realise the larger objective of diversification of economy and empowering private sector to play bigger role. In regard to the question as to how far this role happens to be credible and coherent with the new initiative, only time will tell.

## Limitations of the study

The paper is a critical review of the Healthcare HRD reform adopted in Saudi Arabia in the recent past, specifically Vision 2030 and NTP 2020, which were both adopted in 2016 based on the findings of the MGI report. Saudization (localisation) of the workforce in healthcare sector and their learning and development form an important part of the government policy in Saudi Arabia, and earlier research and insights available on the Saudi healthcare reform were also referred to in that regard. There is a limitation of data to evaluate the efficacy of these provisions adopted under the NPTs in the form of KPIs and KPTs, but an attempt has been made to document and evaluate these provisions from the available resource as of today.

## Abbreviations

HRD: Human Resource Development
KPIs: Key performance indicators
KPTs: Key performance targets
KSA: Kingdom of Saudi Arabia
MGI: McKinsey Global Institute
MOH: Ministry of Health
NTP: National Transformation Program
UN: United Nations
WHO: World Health Organization

## References

[1] Ministry of Health. Annual statistical book. Riyadh: Ministry of Health; 2017. [cited 2018 Sep 27]. Available from: https://www.moh.gov.sa/en/Ministry/Statistics/book/Documents/ANNUAL-STATISTICAL-BOOK-1438H.pdf.

[2] Almalki M, FitzGerald G, Clark M (2011). Health care system in Saudi Arabia: an overview. East Mediterr Health J.

[3] Saudi Arabian Monetary Authority. Fifty fourth annual report. Riyadh: Saudi Arabian Monetary Authority; 2018. [cited 2018 Sep 3]. Available from: http://www.sama.gov.sa/en-US/EconomicReports/AnnualReport/Fifty%20Fourth%20Annual%20Report.pdf.

[4] World Health Organization. The World Health Report 2000: health systems: improving performance. Geneva: World Health Organization 2000.

[5] Al-Hanawi MK (2017). The healthcare system in Saudi Arabia: how can we best move forward with funding to protect equitable and accessible care for all?. Int J Healthcare.

[6] Al-Hanawi MK, Alsharqi O, Almazrou S (2018). Healthcare finance in the Kingdom of Saudi Arabia: a qualitative study of householders’ attitudes. Appl Health Econ Health Policy.

[7] Al-Hanawi MK, Vaidya K, Alsharqi O, Onwujekwe O (2018). Investigating the willingness to pay for a contributory National Health Insurance Scheme in Saudi Arabia: a cross-sectional stated preference approach. Appl Health Econ Health Policy.

[8] United Nations (2004). World population by 2300.

[9] Barrage G, Perillieux R, Shediac R (2007). Investing in the Saudi Arabian healthcare sector.

[10] Saudi Arabia’s Vision 2030. National transformation program 2020. [cited 2018 Aug 11]. 2016. Available from: http://vision2030.gov.sa/sites/default/files/NTP_En.pdf

[11] Al-Borie MH, Abdullah MT (2013). A “DIRE” needs orientation to Saudi health services leadership. Leadersh Health Serv.

[12] Sajjad R, Qureshi M. An assessment of the healthcare services in the Kingdom of Saudi Arabia: an analysis of the old, current, and future systems. Int J Healthc Manag. 2018:1–9.

[13] Sebai ZA, Milaat WA, Al-Zulaibani AA (2001). Health care services in Saudi Arabia: past, present and future. J family Community med.

[14] Al Yousuf M, Akerele T, Al MY (2002). Organization of the Saudi health system. East Mediterr Health J.

[15] McKinsey Global Institute Report. Saudi Arabia beyond oil: the investment and productivity transformation: McKinsey global institute; 2015.

[16] Bronfman J. Health insurance choice, moral hazard and adverse selection: a study of the Chilean case using panel data. 2011. [Cited 2018 Aug 5]. Available from: https://www.american.edu/spa/publicpurpose/upload/Fixed-Submission.pdf

[17] Smith PC (2012). What is the scope for health system efficiency gains and how can they be achieved?. Eurohealth.

[18] Mosca I (2012). Evaluating reforms in the Netherlands’ competitive health insurance system. Eurohealth.

[19] Ahmed M. Saudi Arabia looks to the private sector to meet growing healthcare demands. 2017. [Cited 2018 Sep 27]. Available from: https://www.forbesmiddleeast.com/en/saudi-arabia-looks-to-the-private-sector-to-meet-growing-healthcare-demands/

[20] Al-Nozha MM, Abdullah M, Arafah MR, Khalil MZ, Khan NB, Al-Mazrou YY, Al-Maatouq MA, Al-Marzouki K, Al-Khadra A, Nouh MS, Al-Harthi SS (2007). Hypertension in Saudi Arabia. Saudi Med J.

[21] World Health Organization. Country cooperation strategy for WHO and Saudi Arabia 2012: Regional Office for the Eastern Mediterranean: World Health Organization; 2016. Available from: http://apps.who.int/iris/bitstream/10665/113227/1/CCS_Saudia_2013_EN_14914.pdf

[22] World Health Organization. Non-communicable diseases (NCD) country profiles. Geneva: WHO Press; 2018. Available from: http://www.who.int/nmh/countries/sau_en.pdf.

[23] Al Ghobain MO, Al Moamary MS, Al Shehri SN, AL-Hajjaj MS (2011). Prevalence and characteristics of cigarette smoking among 16 to 18 years old boys and girls in Saudi Arabia. Ann Thorac Med.

[24] Al-Gelban KS (2007). Depression, anxiety and stress among Saudi adolescent school boys. J R Soc Promot Heal.

[25] Mansuri FA, Al-Zalabani AH, Zalat MM, Qabshawi RI (2015). Road safety and road traffic accidents in Saudi Arabia: a systematic review of existing evidence. Saudi Med J.

[26] Almalki A, Al-Hanawi MK (2018). Public private partnerships and collaboration in the health sector in the Kingdom of Saudi Arabia: a qualitative study. Global J Health Sci.

[27] Al-Asfour A, Khan SA (2014). Workforce localization in the Kingdom of Saudi Arabia: issues and challenges. Hum Resour Dev Int.

[28] Mellahi K, Wood G (2002). Desperately seeking stability: the making of the Saudi Arabian growth regime. Compet Chang.

